# Which fatigue scale should I use? A Rasch analysis of two fatigue scales in inflammatory conditions

**DOI:** 10.1093/rheumatology/kead667

**Published:** 2023-12-09

**Authors:** Emerson J Bartholomew, Oleg N Medvedev, Keith J Petrie, Trudie Chalder

**Affiliations:** Department of Psychological Medicine, University of Auckland, Auckland, New Zealand; School of Psychology, University of Waikato, Hamilton, New Zealand; Department of Psychological Medicine, University of Auckland, Auckland, New Zealand; Department of Psychological Medicine, King’s College London, London, United Kingdom

**Keywords:** fatigue, patient-reported outcome measures, inflammatory conditions, Rasch analysis

## Abstract

**Objectives:**

This study aimed to assess the psychometric properties of two widely used fatigue scales in a sample of patients with inflammatory conditions.

**Methods:**

Rasch analysis was used to examine scale reliability, item bias, unidimensionality and overall fit to the Rasch model. Subtest methodology was utilized to attempt to improve model fit for the Chalder Fatigue Scale (CFQ) and Bristol Rheumatoid Arthritis Fatigue Multidimensional Questionnaire (BRAF-MDQ).

**Results:**

Initial analysis displayed strong reliability (person separation index 0.89–0.96) alongside a lack of item bias in both scales. However, evidence for unidimensionality was not found for either scale. Overall fit to the Rasch model was marginal for the CFQ and misfitting for the BRAF-MDQ. Local dependency was observed as well as significant item misfit for both scales. Subtest modifications resulted in the best model fit for the BRAF-MDQ [χ^2^(16) = 15.77, *P* = 0.469] and the CFQ [χ^2^(25) = 15.49, *P* = 0.929]. Modifications resulted in improved fit, reductions in measurement error and the production of ordinal-to-interval conversion tables for both scales. Conversion tables apply the benefits of enhanced measurement accuracy, valid comparison of BRAF-MDQ and CFQ scores to other interval-level data, appropriate use in parametric statistics and enhanced precision in clinical cut-off scores—without the need to change administration format.

**Conclusion:**

The BRAF-MDQ and CFQ are valid, reliable tools for fatigue assessment. Psychometric indices and content factors suggest the CFQ is suited to measuring general fatigue, particularly when response burden is a concern, while the BRAF-MDQ should be used in clinical presentations where other symptoms are severe and the impact of fatigue on daily living and emotional and social well-being is of interest.

Rheumatology key messages

- Both scales are reliable for clinical settings and their suitability depends on assessment aims and practical constraints.
- Rasch analysis revealed issues with local response dependency.
- Subtest modifications resulted in fit to the Rasch model and improvements to scale accuracy.

## Introduction

Fatigue is a pervasive and debilitating symptom prevalent across a range of inflammatory conditions, i.e. ankylosing spondylitis (AS) (45%) [1], rheumatoid arthritis (RA) (40–70%) [2], multiple sclerosis (MS; 68%) [3] and systemic lupus erythematosus (SLE) (67–90%) [4]. The United States (US) National Institute of Medicine defines fatigue as ‘a marked reduction in the ability to continue activities previously engaged with, unrelieved by rest, for a period of six months or longer’ [5]. Patient-reported outcome measures (PROMs) are central to finding a way forward in improving clinical outcomes [8], and this attention has been greatly renewed due to COVID-19 and physical distancing mandates. The Chalder fatigue scale (CFQ [6]) and the Bristol Rheumatoid Arthritis Fatigue Multidimensional Questionnaire (BRAF-MDQ [7]) are both commonly used for research and clinical practice in inflammatory conditions. However, these scales have typically been examined under classical test theory (CTT) approaches (i.e. exploratory and confirmatory factor analysis). CTT methods work under the assumption that an observed item score is equal to a respondent’s true score minus error. While CTT has advantages in its popularity and ease of application, it has several disadvantages, including sample dependency [9], oversimplification of error variability [10] and unknown contributions of items to the latent trait [11], among other issues [12]. Such limitations can be overcome by modern test theory (MTT) approaches, which view observed scores as a function of item difficulty and respondent ability. MTT methods such as Rasch analysis are able to evaluate the individual contributions of each item to a latent trait, as well as establish measurement invariance [13].

The CFQ is a rapid and reliable measure of fatigue validated in community settings as well as in clinical samples (i.e. chronic fatigue, MS and cancer) [6, 14]. CFQ scores are commonly used as outcome measures in research on inflammatory conditions [15, 16]. Cronbach’s α scores show good reliability (α = 0.88) [17–19], and the scale has been widely validated in other countries (e.g. China and Norway) [20, 21]. The CFQ was found to be robust compared with longer and multidimensional fatigue measures [22]. However, the results of these validation studies leave several key issues unresolved. Morriss *et al.* [19] found a four-factor structure in its principal components analysis (PCA), compared with the two-factor structure in the original study [6]. Subsequent investigations show further conflict, with a 2010 PCA in support of a two-factor structure [14] and a 2016 confirmatory factor analysis (CFA) concluding unidimensionality [17].

The BRAF-MDQ is a 21-item scale developed in response to research indicating that fatigue in rheumatological conditions may be a multidimensional construct [7]. The scale is frequently utilized in research on rheumatic conditions [15, 23] and has demonstrated strong reliability indices, however, the factor structure is contested [24]. Two validation studies corroborated a four-factor structure [25, 26]. Conversely, a 2015 MTT analysis found evidence for unidimensionality [27]. This analysis examined areas common to MTT, e.g. differential item functioning (DIF) and global model fit, representing an extension of the methods of the previous studies. However, issues such as local response dependence were not investigated. Recent advances in MTT methods allow for scale modifications that may improve fit indices without the removal of items, endangering content validity [28]. Furthermore, the scale has yet to be converted to an interval level, an advancement possible under Rasch analysis [29].

This study aimed to assess the CFQ and BRAF-MDQ in patients with inflammatory conditions by Rasch analysis. Potential issues such as local response dependency or item biases by age or gender will be examined. Rasch analysis is commonly used in rheumatology research for enhancing or creating PROMs [30, 31]. If a successful fit is achieved, interval conversion can be carried out. Conversion enables appropriate comparisons of BRAF-MDQ and CFQ data with physiological outcome measures relevant to rheumatic conditions. Past investigations have compared CFQ raw scores to interval-level data such as inflammatory cytokine concentrations [32]. However, this is not advised due the unequal distances between response categories present in ordinal data [12]. Additional benefits to interval conversion are the appropriate use of data in parametric tests, as ordinal raw scores are often used in such analyses (i.e. *t*-tests, analysis of variance), violating the assumptions on which such tests operate [12]. Furthermore, scale accuracy issues can have negative effects in the use of imprecise cut-off scores, decreasing their usefulness in aiding diagnosis.

## Methods

### Inclusion criteria

Participants were included if they were >18 years of age and were being seen in a rheumatology outpatient clinic at a teaching hospital in South London.

### Exclusion criteria

Participants who could not read and understand English were excluded, as the measures are validated for English-speaking people only and the validity would be adversely affected if they were to be translated without adherence to strict methodologies.

### Participants

There were 232 participants, with 7 missing responses for age and 2 missing responses for gender. The mean age was 49.18 years (s.d. 14.78) and ages ranged from 17 to 83. The sample included 58 males (25%) and 172 females (74.1%). The sample size is appropriate based on recommendations outlined by Linacre [33]. Three age groups were created (17–40, 41–55 and ≥55) for later item bias testing.

This study was approved by the London Dulwich Research Ethics Committee (REC reference 10/H0808/135).

### Procedure

Data were collected from rheumatology outpatients from November 2011 to March 2013. Participants with autoimmune rheumatic diseases were recruited consecutively from outpatient rheumatology clinics. The study was described to the patients by a researcher or rheumatologist. Those who agreed to take part gave written informed consent and completed self-rating questionnaires in outpatient clinics or at home that were posted by the researcher. Where possible, the diagnoses were provided by a rheumatologist in accordance with respective criteria [34–37].

### Measures

The BRAF-MDQ is a 20-item mixed visual analogue and Likert-type self-report questionnaire developed to measure fatigue in rheumatological conditions, specifically RA [7]. Response categories for item 1 range from 0 ‘no fatigue’ to 10 ‘totally exhausted’, and for item 2 responses range from 0 to 7 days. Item 3 is based on hours, with three response categories from 1 (<1 h) to 3 (12 h). Items 4–20 are measured on a scale of 1–4 scale, where 0 is ‘not at all’ and 3 is ‘very much’.

The CFQ is an 11-item Likert-type self-report measure of fatigue intended for use in both community and clinical settings in conditions such as MS, SLE, chronic fatigue and RA [6, 38]. Items 1–11 are scored on a 0–3 scale from ‘less than usual’ to ‘more than usual’, except for item 11, which goes from ‘better than usual’ to ‘worse than usual’. No items are reverse scored on either the CFQ or BRAF-MDQ.

## Rasch analysis

Analyses were conducted under the partial credit version of the polytomous Rasch model using the RUMM2030 software package version 5.4 (Rummlab, Duncraig, WA, Australia) [39]. Rasch analysis is based on principles outlined by Rasch [40], and extended to polytomous items by Andrich [41], with Masters [42] later establishing the partial credit model. It is an iterative process that aims to achieve fit to the Rasch model assessed through overall and individual item fit statistics. Overall fit uses the item–trait interaction chi-squared value, which should be non-significant. A significant value would indicate that the scale is not functioning appropriately across different levels of the latent trait. Item fit assesses the contribution each item makes to the latent trait, with misfit indicating an inconsistency between the Rasch model and the item. Item fit residuals should range between −2.50 and +2.50. Unidimensionality is tested through the Smith test, with a 5% cut-off value indicating confirmation of unidimensionality [43]. Local dependence refers to a covariation between items that is not attributable to the latent trait and may result in spurious correlations, inflated reliability and false evidence of multidimensionality. Residual correlation matrices are used to determine whether local dependence is present and correlations >0.20 justify modification or removal [44]. Sample targeting assesses how well the scale covers the abilities of the respondents, potentially identifying ceiling or floor effects. Targeting is satisfactory when the sample mean falls between +0.50 and −0.50 in relation to an item mean set to 0. Reliability is assessed by the person separation index (PSI). A value >0.85 indicates appropriate use at the individual level [45]. Measurement invariance, laid out by Thurstone [46] as a fundamental principal of measurement, states that a measurement should not vary depending on what it is measuring. Invariance will be examined through DIF testing by age and sex. DIF notes a situation where individuals from different groups (e.g. males and females) have different probabilities of endorsing an item—even when they have the same latent trait level. Finally, response category thresholds are assessed via item characteristic curves (ICCs). ICCs represent the probability of an individual, possessing a certain amount of a latent factor, selecting a certain response category within an item. Disordered thresholds can undermine person ability and item difficulty parameters.

## Results

### Sample characteristics

A total of 162 participants had RA, seronegative SpA and CTD. The SpA group included the following diagnoses: PsA, seronegative SpA (unspecified), enteropathic arthritis, AS and reactive arthritis. The CTD group included the following diagnoses: CTD (unspecified), SLE, myositis, vasculitis, Behçet’s disease and SS. A total of 70 participants had several comorbid disorders that could not be classified purely as RA, CTD or SpA.

### BRAF-MDQ

Initial analysis displayed high reliability (PSI 0.96) but poor overall fit to the Rasch model indicated by a significant item–trait interaction chi-squared value [χ^2^(60) = 256.38, *P* < 0.01] (Table 1). Item fit residuals showed several instances of significant misfit relating to items 1 (4.05), 2 (6.54) and 5 (4.12), with item 12 showing a marginal misfit (−2.60). Person fit indices showed values within acceptable ranges and stayed this way through to the final analysis (Table 1). The sample mean (−0.30) was slightly lower than the item mean but was at a value within the acceptable cut-off range of ±0.50, yet had a high s.d. (2.1), indicating a possible floor effect. Inspection of disordered thresholds showed dysfunction with category 1, as it never becomes modal.

**Table 1.** Summary of the Rasch model fit statistics for the initial and final analysis of the BRAF-MDQ

Analyses	Item fit residual, mean (s.d.)	Person fit residual, mean (s.d.)	Goodness of fit		PSI^a^	Unidimensionality *t*-test,%
			χ^2^ (df)	*P*-value		
Positive						
Initial	−0.10 (2.43)	−0.18 (1.64)	265.38 (60)	<0.001	0.96	16.59 (no)
Final	0.11 (1.56)	−0.36 (0.93)	15.77 (16)	0.469	0.89	4.59 (yes)

a PSI without extremes.

Evidence of unidimensionality was not found, and local dependence was observed in three clusters: between items 1 and 2, items 8–11 and items 12–16. Subtest methodology was used to attempt to resolve local dependency without removing items in order to preserve content validity [28, 47]. Based on conceptual meaning and residual correlations, four subtests were created by combining items to reflect the BRAF-MDQ subscales. Subtest 1 ‘Physical’ (items 1–4), subtest 2 ‘Living’ (items 5–11), subtest 3 ‘Cognition’ (items 12–16) and subtest 4 ‘Emotion’ (items 17–20) (see Table 2).

**Figure 1.**
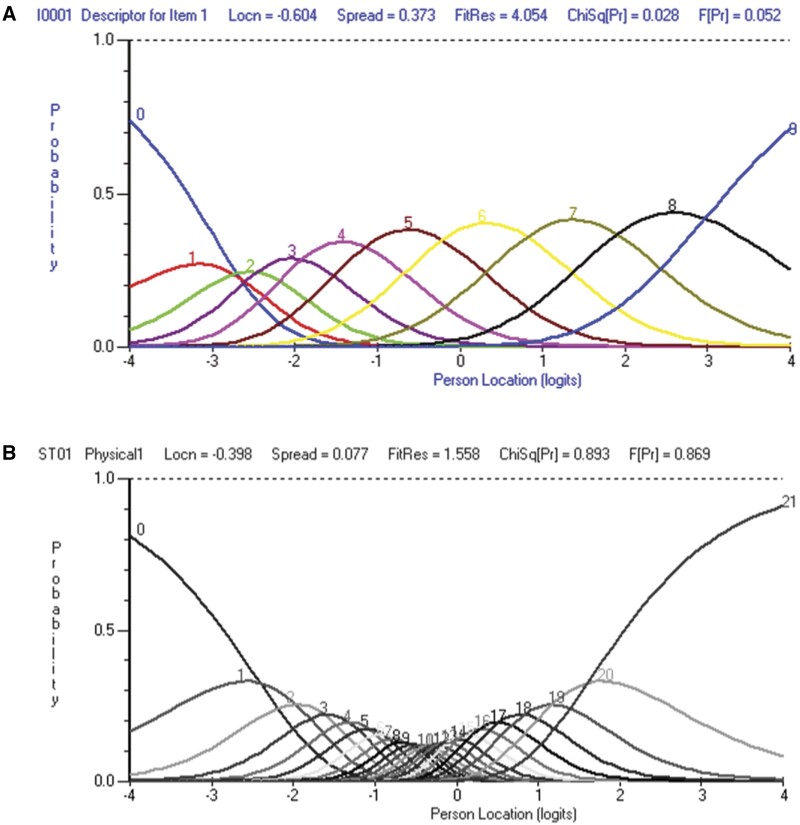
ICCs for item 1 of the BRAF-MDQ **(A)** before and **(B)** after

Modifications resulted in a satisfactory overall fit to the Rasch model with a non-significant item–trait interaction chi-squared value [χ^2^(16) = 15.77, *P* = 0.469], indicating that the items were working well across all latent trait levels. Reliability was maintained at an excellent level (0.89), suitable for use with groups and in individual clinical assessment. Item fit residuals showed no misfit among subtests. Scale targeting improved, as the sample mean and s.d. were reduced, with no evidence of significant floor or ceiling effects (Fig. 1A and B). Disordered threshold category 0 was rectified as seen in Supplementary Fig. S1, available at *Rheumatology* online. Evidence of unidimensionality was observed, with the Smith test showing the lower bound of *t*-tests (4.59%) beneath the 5% cut-off [43]. Issues of local dependency were resolved with no items above the 0.20 cut-off. Modifications showed the best model fit without resorting to the removal of items. DIF testing returned no significant item bias by age or gender.

**Table 2.** Initial (above) and adjusted (below) item fit statistics for the BRAF-MDQ

Item no.	Item	Location	Fit residual	χ^2^
1	Circle (1–10) your average level of fatigue during the past week.	−0.60	4.05	9.13
2	How many days did you experience fatigue during the past week?	−1.34	6.54	174.23
3	How long on average has each episode of fatigue lasted?	−0.34	2.18	9.31
4	Have you lacked physical energy because of fatigue?	−1.12	−1.11	7.44
5	Has fatigue made it difficult for you to have a bath or shower?	1.26	4.12*	2.80
6	I feel very connected to all living things and the earth.	1.31	0.09	2.52
7	Has fatigue made it difficult to dress yourself?	−0.38	−0.95	6.57
8	Has fatigue made it difficult to do your work or other daily activities?	−0.33	0.15	4.51
9	Have you avoided making plans because of fatigue?	−0.42	−2.29	8.83
10	Has fatigue affected your social life?	−0.09	−0.83	2.91
11	Have you cancelled plans because of fatigue?	0.27	−0.88	4.30
12	Have you refused invitations because of fatigue?	0.04	−2.60*	6.45
13	Have you lacked mental energy because of fatigue?	0.22	−0.31	1.49
14	Have you forgotten things because of fatigue?	0.38	−1.10	3.50
15	Has fatigue made it difficult to think clearly?	−0.10	−1.58	3.34
16	Has fatigue made it difficult to concentrate?	0.63	−1.25	1.43
17	Have you made mistakes because of fatigue?	0.00	−2.30	4.95
18	Have you felt less control in areas of your life because of fatigue?	0.83	−1.76	2.89
19	Have you felt embarrassed because of fatigue?	−0.13	−0.71	7.03
20	Has being fatigued upset you?	−0.60	4.05*	9.13
21	Have you felt down or depressed because of fatigue?	−0.09	−1.42	1.77

	Final item modification fit statistics for the BRAF-MDQ			

1	Subtest 1 Physical (1–4)	−0.40	1.56	3.49
2	Subtest 2 Living (5–11)	0.17	0.49	5.75
3	Subtest 3 Cognition (12–16)	0.12	0.53	6.16
4	Subtest 4 Emotion (17–20)	0.11	−2.11	23.03

* Significant misfit at *P* < 0.05.

### CFQ

Initial CFQ analysis showed an acceptable overall model fit with a marginally non-significant item–trait chi-squared statistic [χ^2^(33) = 48.06, *P* = 0.044). Initial reliability was excellent with a PSI of 0.89. Person fit values fell within desirable ranges. No evidence of DIF by age or gender and no disordered thresholds were observed. Sample targeting indicated a ceiling effect, with a sample mean (0.84) greater than the item mean and falling outside the acceptable cut-off of ±0.50. Furthermore, significant item misfit was observed with item 5 (−2.96) and unidimensionality was not found (16.02%) (Table 3).

**Table 3.** Summary of the Rasch model fit statistics for the initial and final analysis of the CFQ

Analyses	Item fit residual, mean (s.d.)	Person fit residual, mean (s.d.)	Goodness of fit		PSI^a^	Unidimensionality *t-*test,%
			χ^2^ (df)	*P*-value		
Positive						
Initial	−0.47 (1.54)	−0.65 (1.69)	48.06 (33)	0.044	0.89	16.02 (no)
Final	0.01(1.90)	−0.56 (1.37)	15.49 (25)	0.929	0.91	3.90 (yes)

a PSI without extremes.

To further investigate unidimensionality, local dependency was assessed and residual correlations were found between items 1 and 3, 2 and 5, 6 and 7, and 9 and 10. Subtest modifications were attempted to resolve local dependence based on both conceptual meaning and residual correlations. Four subtests were created: subtest 1 ‘Sleep’ (items 1 and 3), subtest 2 ‘Physical drain’ (items 9 and 6), subtest 3 ‘Energy’ (items 2, 5 and 10) and subtest 4 ‘Mental drain’ (items 4, 7, 8 and 11).

The item response category characteristic curve shows the likelihood of respondents selecting a certain score on the scale at various levels of the latent trait. An item is better at discriminating between individuals when the curves are peaked and dispersed across all levels of the latent trait. Subtest creation resulted in the best model fit (see Supplementary Fig. S2, available at *Rheumatology* online), with an improved item–trait interaction chi-squared and increased significance value [χ^2^(25) = 15.49, *P* = 0.929], corresponding to a reduction in error stemming from interactions between items and the latent trait. Reliability increased slightly and was above the threshold for appropriate use in individual/clinical assessment. Item misfit was not observed (Table 4), however, targeting showed the ceiling effect remained. Local dependence was not observed and the results of the Smith test confirmed unidimensionality (3.90%). The final person and item distribution is shown in Supplementary Fig. S3, available at *Rheumatology* online.

**Table 4.** Initial items fit statistics for the CFQ

Item no.	Item	Location	Fit residual	χ^2^
1	Do you have problems with tiredness?	−0.82	−0.65	4.85
2	Do you need to rest more?	−0.51	−1.92	5.21
3	Do you feel sleepy or drowsy?	0.02	−0.82	2.20
4	Do you have problems starting things?	0.32	−0.07	2.64
5	Do you lack energy?	−0.48	−2.96*	5.29
6	Do you have less strength in your muscles?	−0.62	0.48	2.02
7	Do you feel weak?	0.06	−1.31	0.63
8	Do you have difficulties concentrating?	0.54	−2.11	4.40
9	Do you make slips of the tongue when speaking?	0.78	1.79	10.40
10	Do you find it more difficult to find the right word?	0.61	1.22	7.24
11	How is your memory?	0.10	1.22	3.20

	Final item modification fit statistics for the CFQ			

1	Subtest 1 (1, 3) Sleep	0.57	−0.80	4.21
2	Subtest 2 (9, 6) Physical drain	0.71	−0.36	2.30
3	Subtest 3 (2, 5, 10) Energy	−1.58	0.87	7.35
4	Subtest 4 (4, 7, 8, 11) Mental drain	0.30	−1.80	15.45

* Significant misfit *P* < 0.05.

Ordinal-to-interval conversion for both the BRAF-MDQ and CFQ is provided in Table 5. This straightforward method converts raw scores from an ordinal to an interval level. Using conversion tables produces improvements to measurement accuracy in accordance with the fundamental principles of measurement—a significant advantage when considering the frequent use of fatigue scores in clinical decision making and analyses via parametric statistics.

**Table 5.** Ordinal-to-interval conversion tables for the BRAF-MDQ and the CFQ

BRAF-MDQ		
Ordinal score	Interval scores	
	Logits	Scale
0	−3.39	0.00
1	−2.74	7.37
2	−2.29	12.34
3	−2.00	15.71
4	−1.77	18.29
5	−1.58	20.38
6	−1.43	22.10
7	−1.30	23.57
8	−1.19	24.82
9	−1.09	25.91
10	−1.01	26.85
11	−0.93	27.70
12	−0.87	28.47
13	−0.80	29.16
14	−0.75	29.80
15	−0.69	30.40
16	−0.65	30.95
17	−0.60	31.47
18	−0.56	31.96
19	−0.51	32.44
20	−0.47	32.90
21	−0.43	33.35
22	−0.39	33.78
23	−0.36	34.21
24	−0.32	34.63
25	−0.28	35.03
26	−0.25	35.44
27	−0.21	35.83
28	−0.18	36.22
29	−0.14	36.60
30	−0.11	36.99
31	−0.08	37.36
32	−0.04	37.73
33	−0.01	38.10
34	0.02	38.46
35	0.05	38.81
36	0.08	39.17
37	0.11	39.51
38	0.15	39.86
39	0.18	40.20
40	0.21	40.55
41	0.24	40.89
42	0.27	41.23
43	0.30	41.56
44	0.33	41.89
45	0.36	42.23
46	0.39	42.57
47	0.42	42.92
48	0.45	43.26
49	0.48	43.61
50	0.51	43.97
51	0.54	44.33
52	0.57	44.70
53	0.61	45.09
54	0.64	45.48
55	0.68	45.89
56	0.72	46.32
57	0.76	46.76
58	0.80	47.23
59	0.84	47.74
60	0.89	48.28
61	0.95	48.89
62	1.01	49.57
63	1.07	50.33
64	1.15	51.25
65	1.25	52.35
66	1.38	53.75
67	1.54	55.61
68	1.78	58.29
69	2.17	62.71
70	2.82	70.00

CFQ		
Ordinal score	Interval scores	
	Logits	Scale
0	−8.49	0.00
1	−7.32	2.66
2	−6.33	4.90
3	−5.50	6.78
4	−4.80	8.39
5	−4.17	9.80
6	−3.61	11.09
7	−3.08	12.28
8	−2.60	13.38
9	−2.14	14.42
10	−1.70	15.42
11	−1.28	16.38
12	−0.88	17.28
13	−0.51	18.13
14	−0.16	18.92
15	0.16	19.66
16	0.47	20.35
17	0.75	20.99
18	1.02	21.60
19	1.27	22.17
20	1.51	22.72
21	1.74	23.25
22	1.97	23.77
23	2.19	24.27
24	2.42	24.79
25	2.65	25.31
26	2.89	25.86
27	3.15	26.44
28	3.43	27.08
29	3.74	27.78
30	4.10	28.61
31	4.55	29.62
32	5.17	31.03
33	6.03	33.00

This conversion table can only be used for respondents with no missing data.

## Discussion

The current article evaluated by Rasch analysis the BRAF-MDQ and the CFQ in a sample of outpatients with inflammatory conditions. Initial results showed both scales misfitting the Rasch model, with no evidence of unidimensionality. Instances of local response dependency were evident and resolved by subtest modifications that resulted in the best fit model, along with evidence of unidimensionality. No item bias by gender or age was observed. Modifications did not involve item removal, which is a danger to content validity. Improvements in model fit correspond to reductions in measurement error and suitability of scale scores to parametric statistics or in comparison with interval-level data. Both the BRAF-MDQ and the CFQ are highly reliable, unidimensional and possess measurement invariance by age and gender. In light of psychometric results and content features, the CFQ is suitable for rapid screening of general fatigue burden in community populations as well as less severe clinical presentations. The BRAF-MDQ is appropriate when time constraints or respondent burden are less concerning. It is suitable for acute clinical fatigue assessment, particularly when more specific aspects of the burden of fatigue on emotional well-being, social life and daily living are required.

Initial analysis revealed poor fit of the BRAF-MDQ to the Rasch model and an absence of evidence for unidimensionality (Table 1). Four misfitting items were observed (items 1, 2, 5 and 12) along with disordered response category thresholds for item 1 (Table 2). Dimensionality was further investigated by examining residual correlations, revealing 11 items with local dependency. Previous investigations asserted the multidimensionality of the BRAF-MDQ [25, 26]. Local dependence arises when items covary for other reasons than their contributions to the overarching trait and can give a misleading impression of multidimensionality. Results indicate the scale had biases stemming from interactions between items (local dependence) and between items and the latent trait, signalling a failure of items to discriminate across respondents with different fatigue levels. To attempt to resolve local dependence, four subtests were created reflecting the four subscales of the BRAF-MDQ. Modifications resulted in an excellent overall fit to the Rasch model, in addition to confirmation of unidimensionality, ordered response category thresholds, no item misfit and no local dependence. Observed results suggest the BRAF-MDQ measures a single, distinct latent trait. The potential floor effect observed suggests unsuitability for community samples or where fatigue is suspected to be lower.

Initial CFQ analysis showed a poor overall model fit, one misfitting item (item 5), local dependence in eight items and lack of evidence for unidimensionality. Additionally, sample targeting indicated a ceiling effect. To attempt to resolve local dependency, subtest modifications combined items into four subtests: ‘Sleep’ (items 1 and 3), ‘Physical drain’ (items 9 and 6), ‘Energy’ (items 2, 5 and 10) and ‘Mental drain’ (items 4, 7, 8 and 11). Modifications resulted in the best overall model fit and evidence of unidimensionality along with no signs of item misfit or local dependency. However, the ceiling effect from the initial analysis remained. Given the CFQ’s intended use in both community and clinical settings, the observed targeting issue could be expected and indicative of the scales ideal use in community samples or in clinical settings where patients are suspected to have mild to moderate fatigue and a shorter, more general assessment of fatigue is desired.

Successful subtest modifications were completed without the removal of items, a major advantage over CTT methods that threaten construct validity and complicate scale implementation. A satisfactory model fit permitted the development of conversion tables (Table 5), providing a straightforward means for researchers and clinicians to increase measurement accuracy (Table 5). Converted data possess a rare feature among latent trait measures—‘concatenability’. This refers to data that can be meaningfully added and subtracted, making it suitable for comparisons with physiological measures and appropriate for use in parametric statistics [11]. The relevance of these properties to the measurement of fatigue is consequential. Fatigue is a construct commonly compared with interval-level diagnostics in inflammatory conditions (e.g. 28-item DAS [48], CRP, ESR). Multiple sclerosis disease activity is often assessed via an array of chemokine and protein biomarkers in cerebrospinal fluid [49]. These comparisons must be made meaningful if PROMs are to be of reliable clinical relevance.

The importance of increased precision is apparent. With respect to clinical cut-offs, the CFQ uses a score ≥18 [50]. Table 5 shows that while raw scores suggest a linear increase in fatigue, the interval scores show points 17, 18 and 19 clustering within a range of 1.18. Hence a score of 16 is nearly 40% closer to the cut-off than previously thought. Conversion shows the middle score range is comparatively tighter, so that a difference between a change from 18 to 19 represents much less of a jump in fatigue (0.57 change) than a change from 31 to 32 (1.41 change). This suggests the clinical cut-off of 18 should be re-evaluated in consideration of the interval score distribution—potentially shifting downwards to 17 or 16, given the 3 points of difference between 16 and 18 is, in reality, a difference of only 1.25.

As for the BRAF-MDQ, 6.1% represents the given change for clinically significant worsening [24] (6.1% = 4 raw scores). Interval conversion shows that moving 4 ordinal points from 12 to 18 equates to moving only 3 points (from 29 to 31) on the interval scale, indicating a real change of 2.86% *vs* the intended change threshold of 6.1%. In terms of clinically significant improvement, a change of 17.5% is outlined (12 raw scores). Looking to the interval conversion, we can see that an ordinal change from 15 to 27 is really only 6 points of interval change (8.57%)—one-half lower than intended [24]. Observed disparities show that raw scores can misrepresent a patient’s true change in fatigue level. For instance, the 6.1% threshold for significant worsening is only half as severe in interval scores. Similarly, significant improvements may only be achieving half the progress originally thought. As a result, minimal clinically important change thresholds would need to be considered and may need to be changed.

A limitation of the current study was that it was conducted on a clinical sample of patients with a range of inflammatory conditions but not in other conditions where the CFQ is commonly used, such as in postoperative and post-stroke patients. About one-quarter of participants had comorbid disorders that could not be classified as RA, CTD or SpA. A proportion of them may have had fibromyalgia, given the high prevalence of fibromyalgia in this group of patients (*n* = 50). Future research could look at replicating these findings in different clinical populations. Additionally, cross-cultural validation was not carried out, making results difficult to generalize to those in other nations. However, modern test theory approaches such as the Rasch analysis are more robust in terms of generalizability compared with CTT methods, which are more sample dependent [45].

Alongside the Functional Assessment Of Chronic Illness Therapy – Fatigue (FACIT-F), a measure commonly used in clinical trials, the BRAF-MDQ and the CFQ are valid and reliable assessments of fatigue in those with inflammatory conditions. The CFQ’s fewer scale items and no questions with time-specific recall increases the ease of administration, making it a better choice when speed or respondent burden is a concern. It is also suited for community settings or for those patients with lower to moderate symptoms, and when an assessment of general fatigue will suffice. The BRAF-MDQ is useful in clinical presentations where other symptoms are severe and where fatigue is suspected to be more severe. Both scales are sensitive enough to changes that are appropriate for clinical assessment. Improvements to measurement accuracy can benefit clinicians in the diagnosis of future patients, evaluating risks of impairment, detecting deterioration or evaluating improvements due to treatment. Future studies could assess whether specific scales work better for different inflammatory disorders.

## Supplementary Material

kead667_Supplementary_Data

## Data Availability

The data used in this article will be shared upon reasonable request to the corresponding author.
