# Supplementary material for: Which fatigue scale should I use? A Rasch analysis of two fatigue scales in inflammatory conditions
Source: Rheumatology (Oxford). 2023 Dec 9;64(1):195–203. doi: 10.1093/rheumatology/kead667 (PMC11701316; doi:10.1093/rheumatology/kead667)
Supplement: kead667_Supplementary_Data [file kead667_supplementary_data.docx]

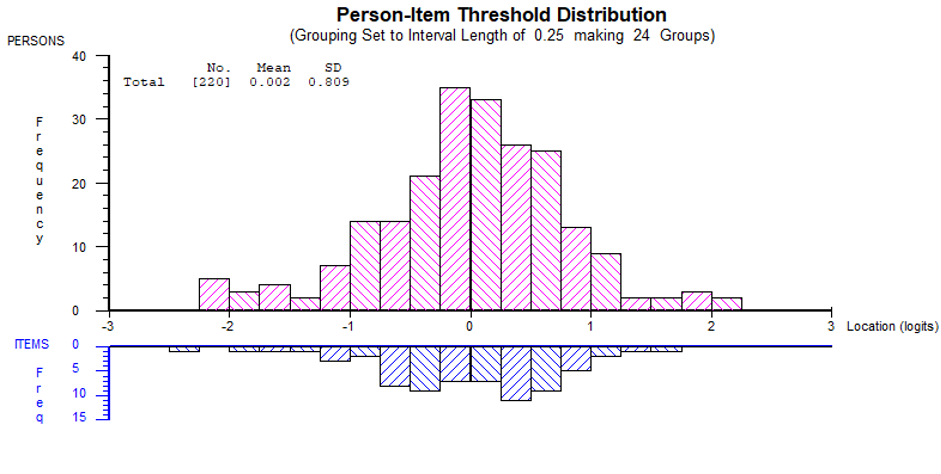


Supplementary Figure S1. *Final analysis person and item distribution thresholds for the BRAF-MDQ*


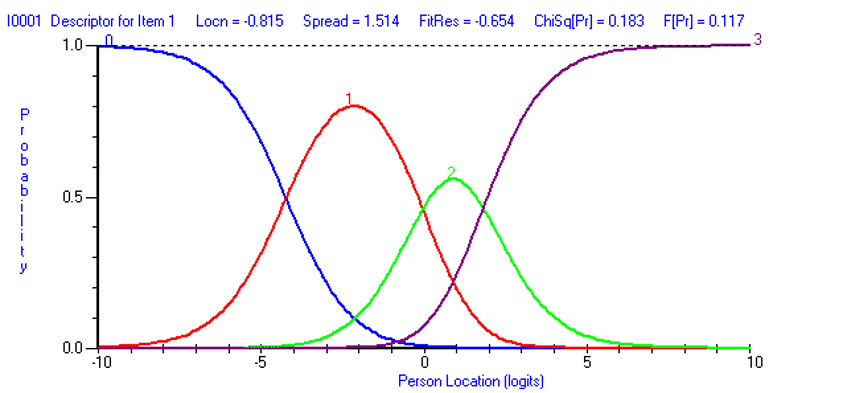


(a)


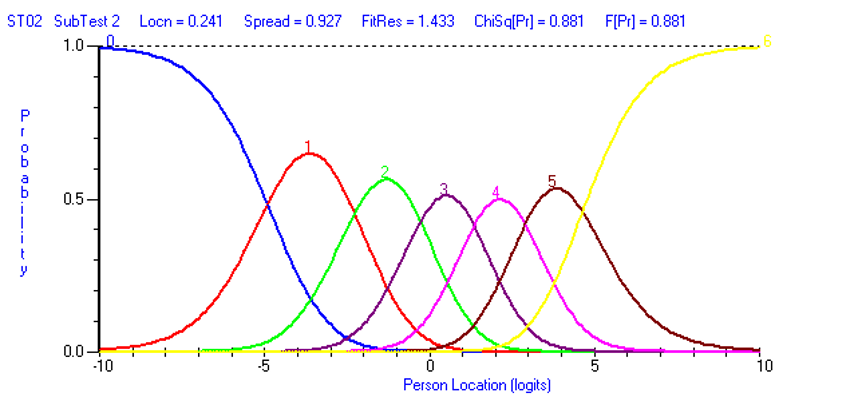


(b)

Supplementary Figure S2. *Item characteristic curve (ICC) for item 1 of the CFQ (a) and subtest 3 (b).*


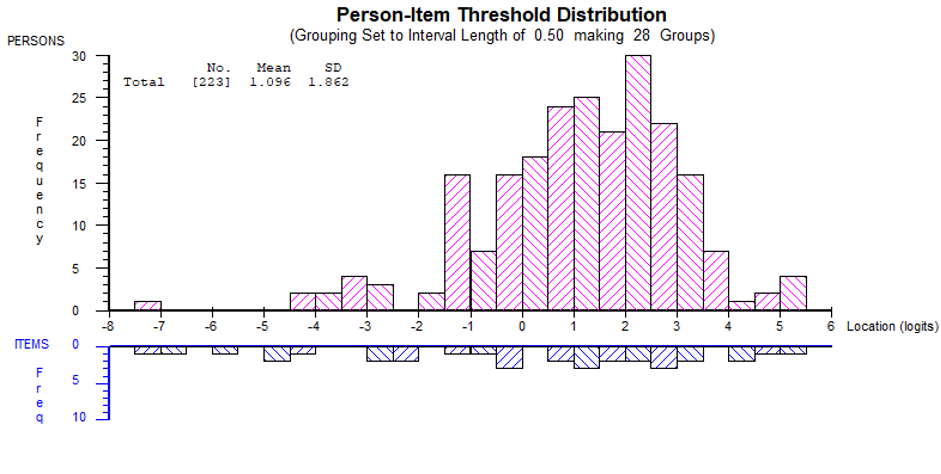


Supplementary Figure S3. *Final analysis person and item distribution thresholds for the CFQ*
